# Machine learning-based lateralization and localization of seizure onset in focal cortical dysplasia patients using ictal scalp EEG

**DOI:** 10.3389/fnins.2026.1915535

**Published:** 2026-07-30

**Authors:** Youmin Shin, Sungeun Hwang, Seung-Bo Lee, Hyoshin Son, Jun-Sang Sunwoo, Sang Kun Lee, Kyung-Il Park, Young-Gon Kim

**Affiliations:** 1 Seoul AI School, Seoul School of Sciences and Technologies University, Seoul, Republic of Korea; 2 Interdisciplinary Program in Bio-engineering, Seoul National University, Seoul, Republic of Korea; 3 Department of Transdisciplinary Medicine, Seoul National University Hospital, Seoul, Republic of Korea; 4 Department of Neurology, Ewha Womans University Mokdong Hospital, Seoul, Republic of Korea; 5 Department of Medical Informatics, Keimyung University School of Medicine, Daegu, Republic of Korea; 6 Department of Neurology, The Catholic University of Korea, Eunpyeong St. Mary’s Hospital, Seoul, Republic of Korea; 7 Department of Neurology, Kangbuk Samsung Hospital, Sungkyunkwan University School of Medicine, Seoul, Republic of Korea; 8 Department of Neurology, Seoul National University College of Medicine, Seoul, Republic of Korea; 9 Department of Neurology, Seoul National University Hospital, Seoul, Republic of Korea; 10 Department of Neurology, Seoul National University Hospital Healthcare System Gangnam Center, Seoul, Republic of Korea; 11 Department of Medicine, Seoul National University College of Medicine, Seoul, Republic of Korea; 12 Healthcare AI Research Institute, Seoul National University Hospital, Seoul, Republic of Korea

**Keywords:** artificial intelligence, epilepsy, focal cortical dysplasia, ictal EEG, machine learning, seizure onset zone

## Abstract

**Inroduction:**

This study aimed to develop and evaluate a machine learning framework for classifying seizure onset zone lateralization and localization in patients with focal cortical dysplasia using ictal scalp electroencephalography (EEG).

**Methods:**

We retrospectively analyzed ictal scalp EEG recordings from 69 patients with focal cortical dysplasia, including 63 surgical and 6 non-surgical patients. EEG signals were preprocessed using common average referencing, segmented into overlapping 1-s windows, and filtered into five frequency bands. Morphological and connectivity features were extracted, and principal component analysis was applied for dimensionality reduction. Automated machine learning was used to select optimal classifiers for lateralization and localization. Model performance was assessed using three-fold cross-validation in 51 surgical patients, internal validation in 12 surgical patients, and extra validation in 6 non-surgical patients.

**Results:**

Before principal component analysis, connectivity features generally outperformed morphological features. Covariance-based connectivity achieved the highest area under the receiver operating characteristic curve for lateralization (0.781), whereas the full connectivity feature set achieved the highest area under the receiver operating characteristic curve for localization (0.786). After principal component analysis, morphology-based energy features showed improved performance, achieving area under the receiver operating characteristic curve values of 0.811 for lateralization and 0.829 for localization in the early post-onset window.

**Discussion:**

These findings suggest that ictal scalp EEG combined with machine learning enables accurate and interpretable seizure onset zone classification in patients with focal cortical dysplasia. The proposed framework may serve as a noninvasive decision-support tool to improve presurgical evaluation and guide invasive EEG planning in focal cortical dysplasia-related epilepsy.

## Introduction

Focal cortical dysplasia (FCD) is one of the most common causes of medically refractory epilepsy (Bast et al., 2006). Characterized by localized malformations in cortical development, FCD often necessitates surgical resection to achieve seizure freedom in refractory cases. The success of epilepsy surgery is critically dependent on precise localization of the seizure onset zone (SOZ), which involves determining both the hemisphere (lateralization) and the anatomical lobe (localization) from which seizures originate (Chakraborty et al., 2020). Traditionally, SOZ localization is achieved through a multi-modal evaluation process involving non-invasive modalities such as scalp EEG, MRI, PET, and SPECT. Intracranial electroencephalography (iEEG) is then employed as a confirmatory tool to refine and validate the presumed seizure focus prior to surgical intervention. While iEEG provides high-resolution spatiotemporal information, it is limited by its partial brain coverage due to the invasive nature of electrode implantation. Furthermore, visual interpretation of EEG recordings by experienced epileptologists is inherently subjective and time-intensive, often requiring years of clinical expertise. In an era of increasing demand for standardized and reproducible clinical tools, the integration of machine learning (ML) into the presurgical evaluation workflow is of growing interest (Natheir, 2021).

While previous studies have demonstrated the feasibility of using interictal EEG for SOZ localization, such approaches rely on indirect biomarkers such as interictal epileptiform discharges (IEDs), connectivity alterations, or spectral imbalances (Varatharajah et al., 2018; Li et al., 2020; Partamian et al., 2025). These interictal markers may provide useful clues but generally reflect the broader irritative zone rather than the SOZ, and their spatial specificity can be limited—especially in FCD, where interictal abnormalities are often diffuse or non-specific. In contrast, ictal EEG reflects the actual electrophysiological dynamics of seizure initiation and provides more direct and specific information about the SOZ. Thus, ictal EEG generally offers greater value for precise localization compared to interictal recordings, particularly in complex pathologies such as FCD.

Recent advances in ML have enabled data-driven approaches to SOZ classification using both interictal and ictal scalp EEG recordings. Numerous studies have employed supervised learning algorithms, such as support vector machines, random forests, and deep neural networks, to infer SOZ lateralization or localization based on either handcrafted features or learned signal representations (Mandhouj et al., 2021; Aboyeji et al., 2025). Interictal-based models often leverage features such as IED rate, high-frequency oscillations, or functional connectivity, whereas ictal-based models focus on time-frequency evolution, spatial propagation, or seizure activity tracking from scalp EEG. Recent deep learning studies have further demonstrated the feasibility of automated seizure activity tracking and onset-zone localization using non-invasive scalp EEG recordings, highlighting the potential value of modeling spatiotemporal ictal propagation (Craley et al., 2022). Despite these promising advances, existing approaches continue to face challenges in interpretability, patient-level generalizability, cohort specificity, and clinical integration. Moreover, many prior models rely on static features or time-averaged representations, potentially overlooking the dynamic nature of ictal activity (Mandhouj et al., 2021; Aboyeji et al., 2025). In contrast, our framework explicitly captures the temporal evolution of frequency-domain patterns by applying time-windowed segmentation and extracting features across multiple frequency bands. This time-resolved approach enables a more nuanced characterization of ictal onset dynamics, enhancing both localization accuracy and clinical interpretability.

To overcome these limitations, we propose a streamlined and interpretable ML framework that leverages ictal scalp EEG segments for SOZ classification in FCD patients. Specifically, the proposed pipeline integrates seizure onset annotations, time-windowed signal segmentation, frequency-domain preprocessing, and both morphological and connectivity-based feature extraction. Dimensionality reduction via principal component analysis (PCA) is used to distill salient signal characteristics, which are then input into tree-based classifiers selected through an AutoML process. This design minimizes manual bias in model selection and enhances generalizability across patients.

In this study, we aim to classify both the lateralization (left vs. right hemisphere) and localization (temporal vs. extratemporal lobe) of the SOZ in patients with FCD. By integrating clinically validated ictal scalp EEG with robust ML strategies, our approach offers a scalable and interpretable solution to presurgical seizure focus identification in a diagnostically challenging population.

## Materials and methods

### Participants and dataset

This study retrospectively analyzed scalp EEG of patients diagnosed with FCD. All consecutive patients who underwent video EEG monitoring as part of the presurgical evaluation between April 2007 and November 2024 at Seoul National University Hospital were screened. EEG data obtained with the Grass Technologies Twin system (Natus Medical, Middleton, WI, USA) were included. Patients with no ictal event during the video EEG monitoring were excluded (*N* = 1). We also excluded patients whose EEG recordings could not be loaded due to corrupted or aborted EDF files (*N* = 6) and those acquired with non-standard EEG systems (*N* = 6). A total of 69 patients were included for this study (Supplementary Figure S1). Among them, 63 patients underwent resective epilepsy surgery and the diagnosis of FCD was pathologically confirmed postoperatively (surgical group). The remaining 6 patients did not undergo surgery after presurgical evaluation, but they were radiologically diagnosed with FCD based on typical MRI findings—cortical thickening, gray–white matter blurring, or transmantle signs—as assessed by neuroradiologists (Widdess-Walsh et al., 2006; Colombo et al., 2003) (non-surgical group). Since the diagnosis of FCD was pathologically confirmed in the surgical group, they included both MRI-positive (MRI suggestive of FCD) and MRI-negative cases.

For model development, the surgically confirmed FCD cohort was divided at the patient level into a 51-patient development cohort and a 12-patient independent internal validation cohort. This split was performed to use the majority of pathologically confirmed cases for model development while preserving an independent surgical validation cohort. Cohort partitioning was performed before seizure segmentation, feature extraction, dimensionality reduction, feature selection, or model training.

The development cohort included 15 patients with left-hemisphere SOZ and 36 with right-hemisphere SOZ; 35 were classified as having temporal lobe epilepsy and 16 as extratemporal lobe epilepsy. An additional 12 surgical patients were assigned to the internal validation set. To further evaluate generalizability, 6 non-surgical patients were included for extra validation. Overall, the dataset comprised MRI-positive cases (development set: 27; internal validation: 8; extra validation: 6) and MRI-negative cases (development set: 24; internal validation: 4) (Table 1; Supplementary Table S1). Because of the limited sample size, MRI status was not explicitly used as a stratification variable during cohort construction. However, the distribution of laterality, localization, and MRI status across the development folds and validation cohorts is provided in Supplementary Table S2. The study was approved by the institutional review board (IRB No. H-2506-098-1649), and the requirement for informed consent was waived due to the retrospective nature of the study.

**Table 1:** Demographic and clinical characteristics of the surgical and non-surgical groups.

Variable	Surgical group (*N* = 63)	Non-surgical group (*N* = 6)	*p*-value
Sex			1.000
Male	36 (57.1%)	3 (50.0%)	
Female	27 (42.9%)	3 (50.0%)	
Onset age (years)	15.5 ± 9.9	16.0 ± 4.9	0.833
Epilepsy duration (years)	10.6 ± 15.4	8.35 ± 12.9	0.696
History of febrile convulsion	14 (22.2%)	0 (0.0%)	0.303
Family history of epilepsy	3 (4.8%)	0 (0.0%)	1.000
MRI-positive	35 (55.6%)	6 (100.0%)	0.074
Laterality of SOZ			1.000
Left	19 (30.2%)	2 (33.3%)	
Right	44 (69.8%)	4 (66.7%)	
Location of SOZ			0.393
Temporal	42 (66.7%)	3 (50.0%)	
Extratemporal	21 (33.3%)	3 (50.0%)	

Values are presented as number (%) or mean ± standard deviation. *p*-values were calculated using Fisher’s exact test for categorical variables and the Mann–Whitney U test for continuous variables. MRI, magnetic resonance imaging; SOZ, seizure onset zone.

### Gold standard for SOZ determination

For the surgical group (*N* = 63), the gold standard for lateralization and localization of SOZ was defined by expert consensus based on multimodal data including EEG, MRI, and when available PET, ictal SPECT, and neuropsychological findings, and confirmed by postoperative histopathological diagnosis. For patients with multiple analyzable seizures, all eligible ictal recordings were included in the analysis, and the same patient-level SOZ label was assigned to all seizures from that patient. For the non-surgical group (*N* = 6), the SOZ lateralization and localization were determined by expert consensus based on typical MRI findings of FCD and concordant interictal and ictal EEG findings.

### EEG preprocessing

All scalp EEG recordings underwent a standardized preprocessing pipeline (Shin et al., 2023; Hwang et al., 2024). EEG data were recorded using the international 10–20 electrode placement system (electrodes: Fp1, F3, C3, P3, Fp2, F4, C4, P4, T1, F7, T7, P7, O1, T2, F8, T8, P8, O2, Fz, Cz, and Pz), with a sampling rate of 400 Hz, a hardware high-pass filter of 0.1 Hz, and a hardware low-pass filter of 500 Hz. The ictal onset for each seizure was determined by an experienced epileptologist and used as the temporal reference point for all subsequent analyses, with seizure onset defined as 0 s. All EEG signals were resampled to 200 Hz before feature extraction using Fourier-method resampling.

Baseline normalization was performed using the 5-s pre-ictal segment from −10 to −5 s relative to seizure onset. For each channel, the mean and standard deviation were calculated from this baseline segment. The signal in each 1-s analysis window was then normalized by subtracting the baseline mean and dividing by the baseline standard deviation. Common average referencing was then applied to reduce spatially shared signal components. Each signal was segmented into 1-s analysis windows using a 0.5-s stride, spanning from −5 to +10 s relative to seizure onset.

Frequency-specific signals were extracted using a fourth-order infinite impulse response Butterworth band-pass filter. Filtering was performed using forward–backward zero-phase filtering. The frequency bands were defined as delta 0.5–4 Hz, theta 4–8 Hz, alpha 8–13 Hz, beta 13–30 Hz, and gamma 30–45 Hz. No additional notch filtering was applied in the computational analysis pipeline. No automated artifact rejection algorithm for muscle artifact, movement artifact, electrode pops, or amplifier saturation was applied after EDF loading. No automated bad-channel detection or channel interpolation procedure was applied. Instead, recordings were excluded during cohort construction if they had no ictal event, could not be loaded because of corrupted or aborted EDF files, or were acquired using non-standard EEG systems.

### Feature extraction

Two main types of features were extracted from each EEG epoch. The first consisted of morphology-based features, which were calculated independently for each channel, frequency band, and 1-s time window. These features included statistical features, Hjorth parameters, and energy-related features. Statistical features included mean absolute amplitude, maximum amplitude, minimum amplitude, standard deviation, skewness, and kurtosis. Hjorth parameters included activity, mobility, and complexity. Hjorth activity was defined as the variance of the signal. Hjorth mobility was calculated as the square root of the variance of the first derivative divided by the variance of the original signal. Hjorth complexity was calculated as the mobility of the first derivative divided by the mobility of the original signal. A small constant was added to denominators where needed to avoid numerical instability (Shin et al., 2023). Energy-related features included total energy, root mean square energy, peak-to-peak amplitude, energy entropy, and log-energy entropy. Total energy was calculated as the sum of squared amplitudes within each analysis window. Root mean square energy was calculated as the square root of the mean squared amplitude. Peak-to-peak amplitude was defined as the difference between the maximum and minimum amplitudes. Energy entropy was calculated from the normalized squared amplitude distribution using base-2 entropy. Log-energy entropy was calculated as the mean logarithm of squared amplitude after adding a small constant to avoid numerical instability.

The second feature domain consisted of connectivity-based features, which were calculated to characterize inter-electrode relationships during ictal evolution. For each frequency band and time window, pairwise inter-electrode Pearson correlation and covariance matrices were computed across the analyzed scalp EEG channels. The upper triangular elements of each matrix, excluding the diagonal, were vectorized and used as connectivity features. Correlation features were used to capture normalized temporal synchrony between electrode pairs, whereas covariance features preserved amplitude-dependent shared variation between electrode pairs (Hwang et al., 2024). Feature extraction was repeated across the five predefined frequency bands and across all peri-ictal time windows. Channel-wise morphology features were concatenated across channels and frequency bands to form the morphology feature set, and pairwise connectivity features were concatenated across electrode pairs and frequency bands to form the connectivity feature set. Zero-crossing features were not included in the final analysis pipeline.

### Dimensionality reduction

To address the high dimensionality of the feature space, principal component analysis (PCA) was applied to reduce the number of input features while retaining the most informative components (Siuly and Li, 2015; Artoni et al., 2018; Saykin et al., 2003). PCA was conducted in three configurations: across the entire dataset (global PCA), within anatomically grouped electrode sets (lobe-wise PCA), and by hemisphere (left/right). The top principal components explaining the majority of variance were retained and used as input features for the ML model. To prevent information leakage, all PCA procedures were performed within the training data only. During three-fold cross-validation, PCA was fitted separately on the training folds and the learned transformation was then applied to the corresponding validation fold.

### Machine learning classification

Two binary classification tasks were formulated: one for lateralization and one for localization. An AutoML approach was employed to develop classification models for seizure lateralization and localization (He et al., 2021; Alsharef et al., 2022). AutoML refers to a suite of techniques that automate the process of model selection, hyperparameter tuning, and performance optimization, reducing the need for extensive manual experimentation. By systematically exploring a wide range of algorithms and configurations, AutoML not only enhances efficiency and reproducibility but also contributes to improved model performance. A range of candidate algorithms was explored within the AutoML framework, including tree-based models such as gradient boosting, random forests, and extremely randomized trees, as well as conventional classifiers such as support vector machines (SVM), k-nearest neighbors (KNN), and logistic regression (Suthaharan, 2016; LaValley, 2008; Rigatti, 2017; Geurts et al., 2006). The optimal model and corresponding hyperparameters were automatically selected based on cross-validation performance within the development dataset. AutoML was used to search across model types and hyperparameters to identify the optimal classifier for each task. No class balancing or resampling was applied.

### Graph-theoretical analysis

To provide a network-level interpretation of task-relevant connectivity patterns, graph-theoretical metrics were computed from band-specific inter-electrode connectivity matrices. Feature importance was estimated using SHAP, a game-theoretic additive feature-attribution method for interpreting model predictions (Lundberg and Lee, 2017). The representative frequency band for each task was selected based on the frequency-band distribution of the top SHAP-ranked connectivity features. Beta-band connectivity was most frequently represented among the important features for lateralization, whereas theta-band connectivity was most prominent for localization. Therefore, average degree centrality, average clustering coefficient, and global efficiency were computed from beta-band connectivity matrices for lateralization and from theta-band connectivity matrices for localization.

### Validation strategy

Model performance was evaluated across three stages: (1) three-fold cross-validation on the 51-patient development dataset, (2) internal validation using 12 patients, and (3) extra validation using six non-surgical patients. All data partitioning was performed at the patient level. For three-fold cross-validation within the development cohort, stratified group k-fold cross-validation was used, with PatientID specified as the grouping variable. Therefore, multiple sessions, seizures, or overlapping epochs derived from the same patient were assigned exclusively to one fold and were never split between training and validation folds. Stratification was performed using the target label for each classification task. The target label was left versus right hemisphere for lateralization and temporal versus extratemporal location for localization.

During model development, the primary evaluation metric used for model selection and hyperparameter tuning within the AutoML framework was the area under the receiver operating characteristic curve (AUC). Feature selection, model fitting, and hyperparameter optimization were performed only within the training fold, and the selected features and fitted model were then applied to the held-out validation fold. Final model performance was assessed using AUC, accuracy, sensitivity, and specificity. This three-tiered validation strategy was designed to evaluate both the robustness of the classification models and their generalizability to independent patient cohorts not involved in model training. The overall analytical workflow, including cohort definition, non-PCA feature analysis, PCA-based feature analysis, and subgroup robustness assessment, is summarized in Supplementary Figure S2.

### Statistical analysis

Baseline demographic and clinical characteristics were compared between the surgical and non-surgical groups. Fisher’s exact test was used for categorical variables, and the Mann–Whitney U test was used for continuous variables. For the main figures and tables, 95% confidence intervals were calculated for model performance metrics. Confidence intervals for AUC were estimated using patient-level bootstrap resampling with 1,000 iterations to preserve the dependency structure among multiple seizures, sessions, and overlapping epochs derived from the same patient. In each bootstrap iteration, patients were sampled with replacement, and all seizures, sessions, and epochs derived from the sampled patients were retained together. The 95% confidence interval was defined as the 2.5th and 97.5th percentiles of the bootstrap distribution. The time-resolved analyses and feature-domain comparisons were considered exploratory model-screening analyses. Therefore, exhaustive pairwise statistical comparisons across all time windows and feature configurations were not performed. Instead, model performance was interpreted using AUC values and their 95% confidence intervals. To explore the relationship between model prediction correctness and postoperative seizure outcome, surgical outcomes were categorized as favorable (Engel class I–II) or unfavorable (Engel class III–IV), and model predictions were categorized as correct or incorrect based on agreement with the expert-defined SOZ. A 2 × 2 contingency table was constructed, with a correct prediction treated as a positive test result and a favorable surgical outcome as the positive reference condition. Accordingly, sensitivity represented the proportion of favorable-outcome patients with correct predictions, specificity represented the proportion of unfavorable-outcome patients with incorrect predictions, and accuracy represented the overall agreement between prediction correctness and surgical outcome.

### Software and reproducibility

All computational analyses were performed using Python 3.8.18 in a Jupyter Notebook environment. EDF files were handled using pyEDFlib 0.1.38. Signal processing, including resampling and Butterworth band-pass filtering, was implemented using SciPy 1.10.1. Numerical computation and data handling were performed using NumPy 1.24.3 and pandas 1.5.3. Dimensionality reduction, feature selection, random forest classification, recursive feature elimination, and stratified group k-fold cross-validation were implemented using scikit-learn 1.2.2. XGBoost models were implemented using xgboost 2.0.3. Model interpretation using SHAP feature importance was performed using SHAP 0.44.1. Graph-theoretical metrics were computed using NetworkX 3.1. Figures were generated using Matplotlib 3.6.0 and Seaborn 0.13.2. Random seeds were fixed at 1209 for model training where applicable.

## Results

Model performance without PCA was evaluated using different combinations of feature types (Table 2). In the lateralization task (left vs. right hemisphere), the highest AUC was achieved when connectivity features based on covariance matrices were used, yielding an AUC of 0.781. For localization (temporal vs. extratemporal), the best performance (AUC = 0.786) was observed when both correlation and covariance features were used together. Across both classification tasks, connectivity-based features consistently outperformed morphology-based features, suggesting that inter-electrode dynamics provide more discriminative power than single-channel morphological characteristics.

**Table 2:** Classification performance across feature types and domains before PCA.

GT	Feature domain	Feature type	AUC [95% CI]
L vs. R	Connectivity	Entire	0.711 ± 0.083 [0.655–0.773]
L vs. R	Connectivity	Correlation	0.698 ± 0.034 [0.676–0.721]
L vs R	Connectivity	Covariance	**0.781** **± 0.072 [0.730–0.838]**
L vs. R	Morphology	Entire	0.658 ± 0.038 [0.633–0.679]
L vs. R	Morphology	Hjorth	0.651 ± 0.066 [0.605–0.698]
L vs. R	Morphology	Energy	0.583 ± 0.037 [0.555–0.601]
L vs. R	Morphology	Statistics	0.665 ± 0.081 [0.601–0.723]
T vs ET	Connectivity	Entire	**0.786 ± 0.093 [0.710–0.855]**
T vs. ET	Connectivity	Correlation	0.762 ± 0.091 [0.695–0.828]
T vs. ET	Connectivity	Covariance	0.751 ± 0.048 [0.718–0.785]
T vs. ET	Morphology	Entire	0.711 ± 0.058 [0.672–0.759]
T vs. ET	Morphology	Hjorth	0.731 ± 0.032 [0.706–0.754]
T vs. ET	Morphology	Energy	0.689 ± 0.058 [0.646–0.732]
T vs. ET	Morphology	Statistics	0.701 ± 0.049 [0.669–0.738]

AUC values are presented as mean ± standard deviation with 95% confidence intervals. Confidence intervals were estimated using patient-level bootstrap resampling. This analysis was performed before PCA-based dimensionality reduction. AUC, area under the receiver operating characteristic curve; CI, confidence interval; ET, extratemporal; L, left; PCA, principal component analysis; R, right; T, temporal. Bold indicate the highest AUC within each GT group.

**Figure 1:**
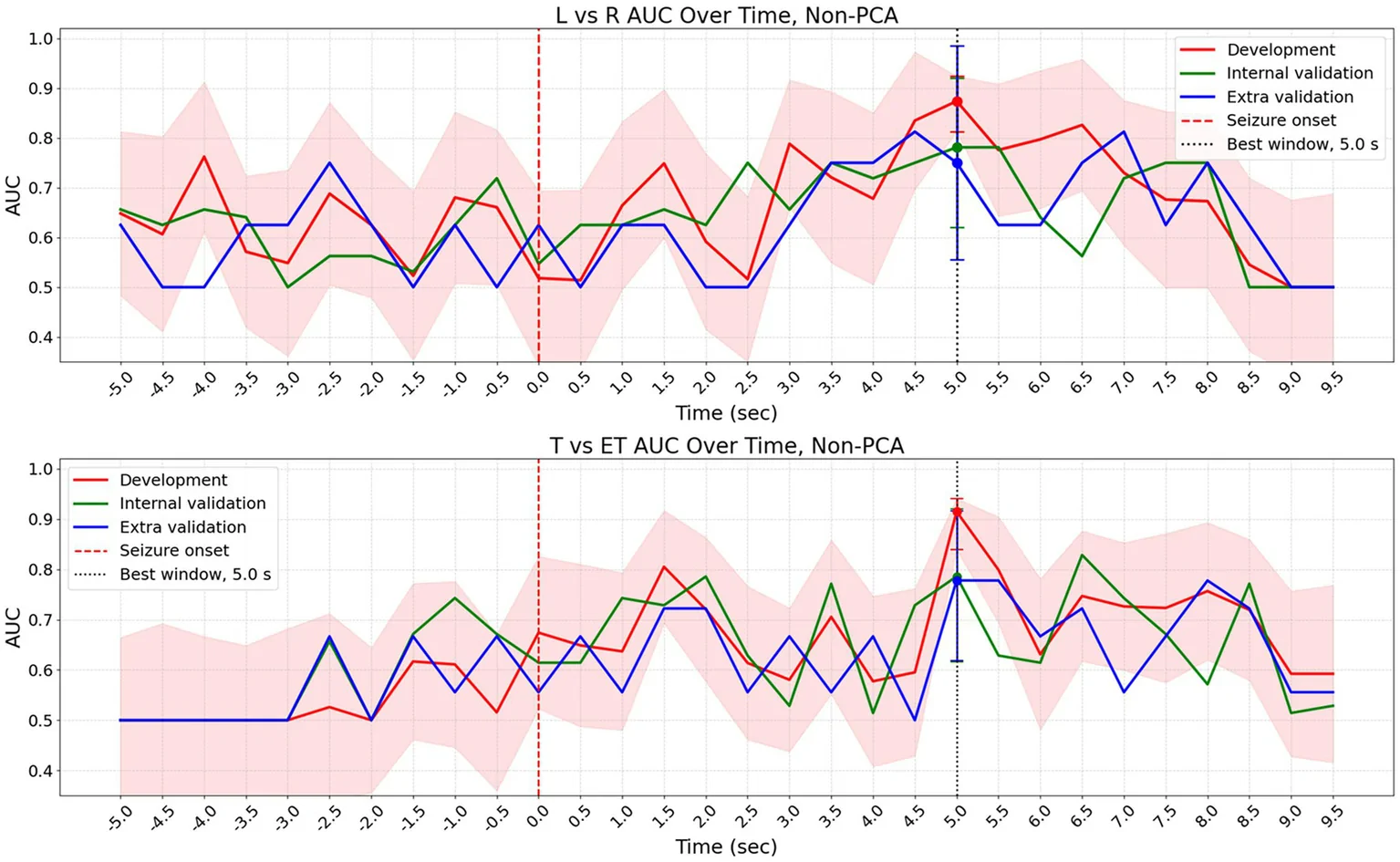
Time-resolved AUC performance of classification models before PCA. Time-resolved AUC values are shown for lateralization (L vs. R) and localization (T vs. ET) across peri-ictal 1-s windows before PCA-based dimensionality reduction. Development, internal validation, and extra validation results are shown as separate curves. The red dashed vertical line indicates seizure onset, and the black dotted vertical line indicates the best-performing window selected from the development set. Error bars at the best-performing window indicate 95% confidence intervals for the corresponding AUC values. AUC, area under the receiver operating characteristic curve; CI, confidence interval; ET, extratemporal; L, left; PCA, principal component analysis; R, right; T, temporal.

For the best feature domain, we performed a time-window screening analysis to identify the most informative peri-ictal temporal interval. Model performance over time was analyzed by computing the AUC within sequential 1-s epochs from −5 to +10 s relative to seizure onset, using the candidate feature set prior to subsequent feature selection. Therefore, the time-resolved AUCs shown in Figure 1 should be interpreted as temporal screening results rather than final optimized model performance.

As shown in Figure 1, both classification tasks—lateralization (left vs. right) and localization (temporal vs. extratemporal)—demonstrated meaningful predictive performance even before ictal onset, indicating that preictal EEG segments contained informative features for classification. However, in both tasks, the highest AUC was consistently observed in the 5.0–6.0 s post-onset, corresponding to the early propagation phase of the seizure. Specifically, the peak AUC for lateralization reached 0.874 [95% CI, 0.813–0.924] in the development set, 0.781 [95% CI, 0.620–0.920] in the internal validation set, and 0.750 [95% CI, 0.555–0.985] in the extra validation set. Performance metrics at the best-performing window are summarized in Supplementary Table S3. Localization achieved maximum AUC values of 0.915 [95% CI, 0.840–0.941] in the development set, 0.786 [95% CI, 0.616–0.920] in the internal validation set, and 0.778 [95% CI, 0.619–0.916] in the extra validation set during this interval.

**Figure 2:**
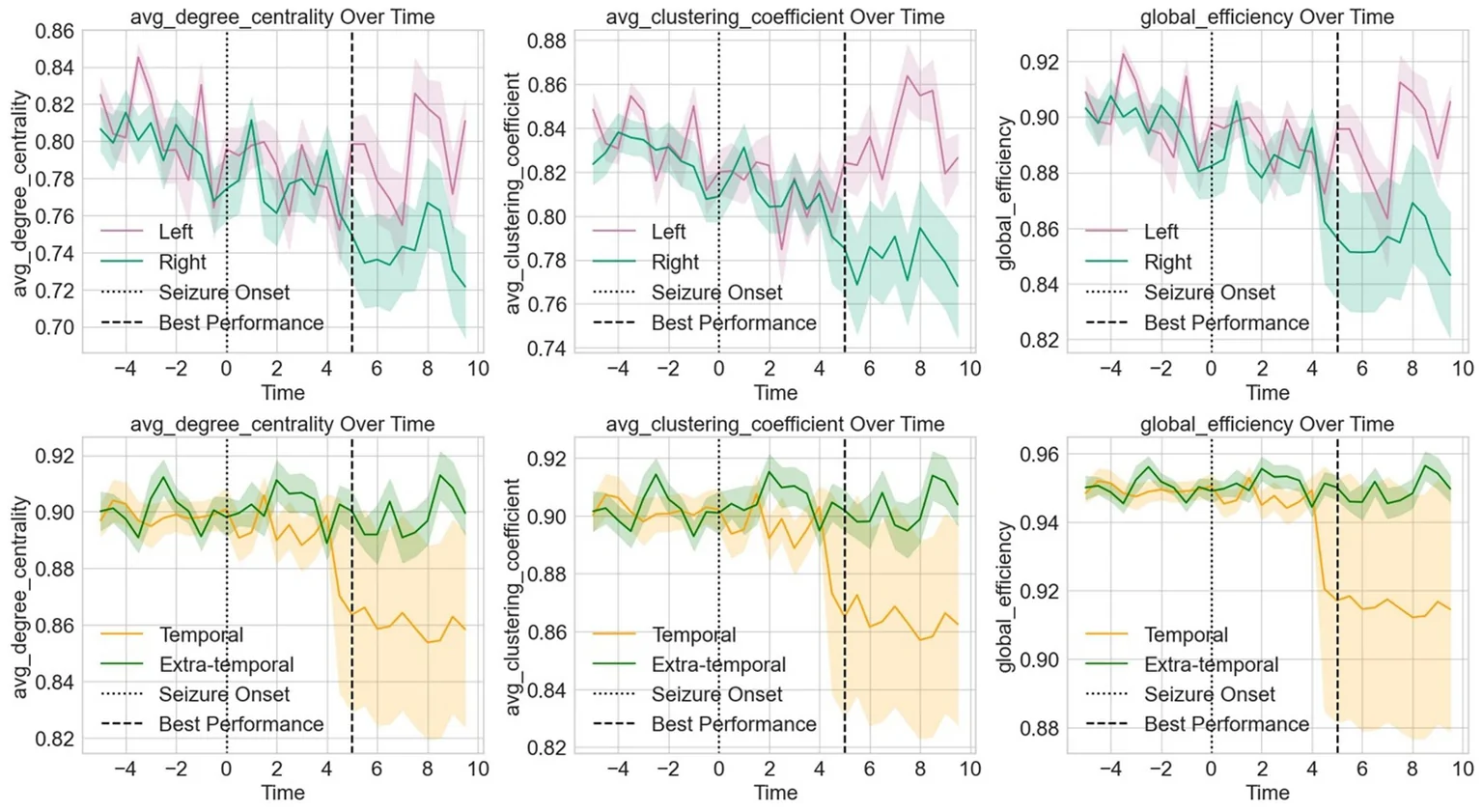
Time-resolved global graph-theoretical connectivity metrics. Time-resolved average degree centrality, average clustering coefficient, and global efficiency are shown for beta-band lateralization networks (top) and theta-band localization networks (bottom). The vertical dotted line indicates seizure onset, and the vertical dashed line indicates the best-performing time window. Shaded areas indicate variability across patients. ET, extratemporal; L, left; R, right; T, temporal.

To provide a network-level interpretation of the best-performing connectivity-based models, global graph-theoretical metrics—including average degree centrality, average clustering coefficient, and global efficiency—were examined (Figure 2). Based on the frequency-band distribution of SHAP-ranked connectivity features, beta-band connectivity was selected as the representative band for lateralization, whereas theta-band connectivity was selected for localization. In the lateralization task, beta-band global network measures tended to be relatively preserved or enhanced after ictal onset in left-hemisphere seizures, whereas right-hemisphere seizures showed more pronounced declines. In the localization task, theta-band global network measures showed a similar pattern of divergence, with extratemporal seizures exhibiting relatively preserved network organization compared with temporal seizures. These findings suggest that ictal connectivity dynamics vary according to SOZ lateralization and localization, and that task-specific frequency bands may provide complementary network-level information for characterizing seizure onset patterns.

Supplementary Table S4 compares multiple PCA strategies—including laterality-wise, lobe-wise, and entire-channel-level decompositions—to evaluate the effect of spatial granularity on classification performance. Entire-channel-level PCA yielded the highest performance for morphology-based models, whereas lobe-wise PCA performed best for lateralization using connectivity features. Because entire-channel-level morphology features achieved the highest overall AUCs, subsequent feature-type analyses were conducted using the entire-channel-level PCA representation. Table 3 then explores the impact of different feature subtypes using this fixed PCA representation. Energy-based morphology features emerged as the most informative, achieving the highest AUCs (0.811 for lateralization and 0.829 for localization), underscoring their enhanced sensitivity to ictal alterations when transformed via PCA.

**Table 3:** Classification performance across feature types and domains after PCA-based dimensionality reduction.

GT	Feature domain	Feature type	AUC [95% CI]
L vs. R	Connectivity	Entire	0.629 ± 0.087 [0.567–0.691]
L vs. R	Connectivity	Correlation	0.711 ± 0.051 [0.675–0.747]
L vs. R	Connectivity	Covariance	0.698 ± 0.098 [0.628–0.768]
L vs. R	Morphology	Entire	0.767 ± 0.058 [0.726–0.808]
L vs. R	Morphology	Hjorth	0.648 ± 0.061 [0.604–0.692]
L vs R	Morphology	Energy	**0.811 ± 0.059 [0.769–0.853]**
L vs. R	Morphology	Statistics	0.807 ± 0.059 [0.765–0.849]
T vs. ET	Connectivity	Entire	0.735 ± 0.080 [0.678–0.792]
T vs. ET	Connectivity	Correlation	0.751 ± 0.068 [0.702–0.800]
T vs. ET	Connectivity	Covariance	0.749 ± 0.037 [0.723–0.775]
T vs. ET	Morphology	Entire	0.796 ± 0.098 [0.726–0.866]
T vs. ET	Morphology	Hjorth	0.760 ± 0.076 [0.706–0.814]
T vs ET	Morphology	Energy	**0.829 ± 0.072 [0.777–0.881]**
T vs. ET	Morphology	Statistics	0.767 ± 0.065 [0.721–0.813]

AUC values are presented as mean ± standard deviation with 95% confidence intervals. Confidence intervals were estimated using patient-level bootstrap resampling. This analysis was performed after PCA-based dimensionality reduction. AUC, area under the receiver operating characteristic curve; CI, confidence interval; ET, extratemporal; L, left; PCA, principal component analysis; R, right; T, temporal. Bold indicate the highest AUC within each GT group.

**Figure 3:**
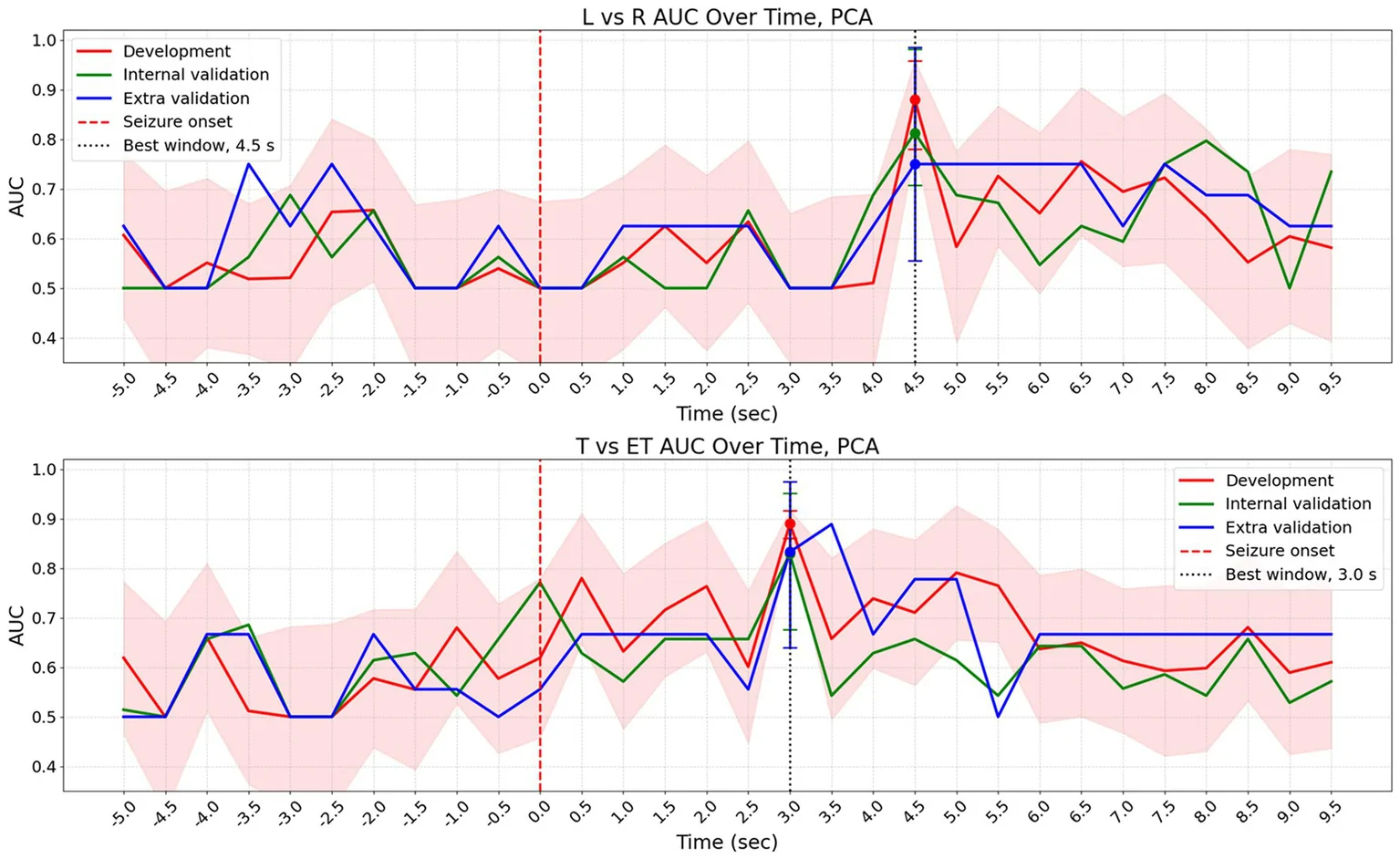
Time-resolved AUC performance of classification models after PCA-based dimensionality reduction. Time-resolved AUC values are shown for lateralization (L vs. R) and localization (T vs. ET) across peri-ictal 1-s windows after PCA-based dimensionality reduction. Development, internal validation, and extra validation results are shown as separate curves. The red dashed vertical line indicates seizure onset, and the black dotted vertical line indicates the best-performing window selected from the development set. Error bars at the best-performing window indicate 95% confidence intervals for the corresponding AUC values. AUC, area under the receiver operating characteristic curve; CI, confidence interval; ET, extratemporal; L, left; PCA, principal component analysis; R, right; T, temporal.

Figure 3 shows the time-resolved AUC of classification models across sequential 1-s windows from −5.0 to +10.0 s relative to seizure onset after PCA-based dimensionality reduction. In both tasks, AUC increased markedly after seizure onset, although the timing of peak performance differed between classification tasks. Peak performance was observed at 4.5 s post-onset for lateralization and at 3.0 s post-onset for localization, suggesting that the most discriminative ictal dynamics emerged during the early post-onset period. These findings suggest that ictal evolution during this early phase provides discriminative signal dynamics that are effectively captured through PCA-reduced morphology features. In the PCA-based temporal screening analysis performed prior to subsequent feature-type optimization, AUCs peaked at 0.880 [95% CI, 0.780–0.958] for lateralization and 0.890 [95% CI, 0.861–0.917] for localization in the development set. In the internal validation and extra validation sets, the corresponding peak AUCs were 0.813 [95% CI, 0.708–0.982] and 0.750 [95% CI, 0.555–0.985] for lateralization, and 0.800 [95% CI, 0.676–0.951] and 0.833 [95% CI, 0.640–0.975] for localization, respectively (Supplementary Table S5).

We further performed subgroup analyses according to MRI status in the internal validation cohort. In the PCA-based models, lateralization performance was comparable between MRI-positive and MRI-negative cases. The AUC was 0.799 ± 0.067 in MRI-positive cases and 0.889 ± 0.096 in MRI-negative cases. For localization, the corresponding AUC values were 0.800 ± 0.176 and 0.792 ± 0.361, respectively. These findings suggest that the proposed ictal scalp EEG-based framework may retain diagnostic performance in MRI-negative FCD cases, although the subgroup sample size was limited and the results should be interpreted as exploratory.

For left–right lateralization, the agreement between model prediction correctness and postoperative seizure outcome yielded an accuracy, sensitivity, and specificity of 0.720 ± 0.033, 0.846 ± 0.030, and 0.852 ± 0.052 without PCA, respectively, and 0.753 ± 0.019, 0.870 ± 0.023, and 0.778 ± 0.000 with PCA. For temporal–extratemporal classification, the corresponding values were 0.733 ± 0.025, 0.846 ± 0.023, and 0.778 ± 0.157 without PCA, and 0.747 ± 0.019, 0.870 ± 0.030, and 0.815 ± 0.052 with PCA. These measures reflect the correspondence between prediction correctness and surgical outcome rather than the primary classification performance of the models.

## Discussion

This study demonstrates the feasibility and clinical utility of ML models trained on ictal scalp EEG data to classify both seizure onset lateralization and localization in patients with FCD. In contrast to conventional approaches that depend heavily on interictal biomarkers or expert-reviewed iEEG, our framework incorporates expert-labeled ictal segments, time-frequency decomposition, and automated model selection, achieving promising performance in both internal and extra validation sets.

Our proposed scalp EEG–based machine learning framework extends prior work by quantitatively analyzing ictal scalp EEG for noninvasive SOZ classification. In conventional presurgical evaluation, expert-interpreted ictal scalp EEG has shown approximately 70% sensitivity in MRI-negative neocortical epilepsy, but its diagnostic value remains dependent on visual interpretation and multimodal clinical concordance (Lee et al., 2005; Conrad et al., 2024; Pilet et al., 2025). More recent machine learning studies have used interictal intracranial EEG for seizure lateralization or SOZ prediction, including spike-rate-based lateralization in temporal lobe epilepsy and functional connectivity–based mapping of SOZ-related contacts (Lee et al., 2005; Conrad et al., 2024; Pilet et al., 2025). Moreover, inter-rater agreement in EEG interpretation remains suboptimal, even among experienced electroencephalographers (Grant et al., 2014; Park et al., 2025). In contrast, our framework focuses on ictal scalp EEG in patients with FCD and performs both SOZ lateralization and localization in a fully noninvasive setting. Using time-resolved feature extraction and PCA-based dimensionality reduction, the temporal screening analysis identified peak development-set AUCs of 0.880 for lateralization and 0.890 for localization, while the final PCA-based energy-feature models achieved AUCs of 0.811 and 0.829, respectively. Although direct comparison with iEEG-based methods is limited by differences in signal modality, spatial resolution, and reference standards, these findings suggest that ictal scalp EEG can provide clinically meaningful adjunctive information for early SOZ estimation and invasive EEG planning in surgical candidates.

A central finding was the strong classification performance achieved using connectivity-based features, particularly inter-electrode covariance matrices, prior to dimensionality reduction. These combinations of features effectively captured large-scale network changes associated with ictal propagation, which are often underrepresented in morphology-based descriptors (Supplementary Figures S3, S4) (Gut et al., 2007; Nicolini et al., 2019; Helmstaedter et al., 1997; Slezicki et al., 2009).

Beyond local inter-electrode interactions, global network organization also revealed meaningful differences across seizure types. In both lateralization and localization tasks, seizures originating from the left hemisphere and extratemporal regions exhibited relatively preserved—or in some cases enhanced—global graph-theoretical metrics, including average degree centrality, clustering coefficient, and global efficiency during the early ictal phase (Cámpora et al., 2019; Lehnertz et al., 2001). These findings suggest that such seizures may engage more distributed or resilient network architectures, enabling the brain to maintain coherent functional integration even in the presence of ictal activity. This interpretation is consistent with the concept that FCD-related epilepsy may involve distributed epileptogenic networks rather than a purely focal lesion, and that electrophysiological abnormalities can extend beyond the MRI-visible lesion (Xu et al., 2025; Gennari et al., 2024). Conversely, right-hemisphere and temporal lobe seizures were marked by greater degradation in global connectivity measures, indicating more severe network disintegration and impaired inter-regional communication. This contrast highlights the complementary value of global connectivity metrics for characterizing seizure onset patterns, and supports their potential as noninvasive EEG-based markers to guide lateralization and localization in presurgical evaluation.

PCA particularly enhanced the discriminative performance of morphology-based features across both classification tasks. While PCA is conventionally applied for dimensionality reduction, in this context it also facilitated the identification of spatially coordinated and frequency-specific EEG patterns that may not be linearly separable in raw feature space (Mikula and Niebur, 2006; Petersen et al., 2016). Notably, the most discriminative components were not the first few principal axes, which often capture global amplitude or noise, but rather mid-level components related to delta- and beta-band energy-related features (Supplementary Figures S5, S6).

Furthermore, time-resolved classification analysis revealed that the early propagation window—between 3 to 6 s after seizure onset—was consistently the most informative in both tasks. This interval likely corresponds to the phase when epileptogenic networks begin to express local propagation dynamics before generalization. The observation that even preictal windows yielded above-chance performance highlights the richness of peri-ictal scalp EEG dynamics and their predictive potential beyond IED detection or resting-state analysis.

Our model also showed preliminary generalizability, maintaining performance in an extra cohort of non-surgical patients whose SOZs were defined by expert consensus using multimodal clinical data. Because this group lacked surgical confirmation, these findings should be interpreted as supportive but not definitive evidence of external applicability. Nevertheless, the consistent trends observed in this cohort suggest that ictal EEG-based classification frameworks may be useful as adjunctive tools in real-world presurgical evaluation, including in patients who do not proceed to surgery. MRI status is clinically important when interpreting the potential role of ictal scalp EEG-based SOZ classification. In MRI-positive FCD, structural lesion information can help constrain the presurgical hypothesis and guide SEEG implantation planning. In contrast, MRI-negative FCD often requires greater reliance on functional and electrophysiological evidence because structural imaging does not provide a clear lesion target. Therefore, a quantitative ictal scalp EEG-based framework may be particularly relevant in MRI-negative cases, where non-invasive localization and SEEG planning remain challenging.

We additionally evaluated model performance in MRI-negative and MRI-positive subgroups using Youden-index thresholds within the internal validation set (Supplementary Table S6). The PCA-based models showed broadly comparable AUC values across MRI-status subgroups. For lateralization, AUC was 0.799 ± 0.067 in MRI-positive cases and 0.889 ± 0.096 in MRI-negative cases. For localization, AUC was 0.800 ± 0.176 in MRI-positive cases and 0.792 ± 0.361 in MRI-negative cases. These findings suggest that the proposed EEG-based framework may have potential utility in MRI-negative FCD. However, this subgroup analysis was exploratory because of the small number of MRI-negative cases in the internal validation cohort. Although stratified group cross-validation was used to preserve patient-level independence and target-label balance, MRI status and other non-target clinical variables were not jointly used as stratification factors because of the limited cohort size. Therefore, residual case-mix imbalance across folds or validation cohorts may have influenced model performance. In particular, MRI-negative cases and extratemporal cases may show more heterogeneous scalp EEG propagation patterns, which could affect both lateralization and localization performance. MRI-status-specific and subgroup-specific performance should therefore be validated in larger, prospectively collected cohorts with balanced MRI-positive and MRI-negative cases, as well as balanced temporal and extratemporal subgroups.

We also performed an exploratory subgroup analysis of left–right lateralization performance according to SOZ location. This analysis was motivated by the clinical possibility that lateralizing ictal scalp EEG patterns may be more readily expressed in temporal lobe seizures than in extratemporal seizures (Chowdhury et al., 2021), although this was not a primary hypothesis of the study. In our data, the temporal subgroup showed higher lateralization performance than the extratemporal subgroup, with higher AUC and accuracy values (Supplementary Table S7). This finding suggests that the proposed model may capture lateralized ictal scalp EEG patterns more effectively in temporal lobe cases, whereas extratemporal seizures may pose greater difficulty because of more variable propagation patterns, broader spatial involvement, or weaker scalp-recorded lateralizing signals. However, given the small subgroup sizes, this analysis should be interpreted as exploratory and hypothesis-generating rather than as definitive evidence of location-dependent model performance.

The proposed framework should be interpreted as a potential adjunctive decision-support tool rather than a replacement for expert multimodal presurgical evaluation. In a possible clinical workflow, the model could be applied after expert annotation of ictal onset to provide quantitative support for SOZ lateralization and broad temporal versus extratemporal localization. Such information may help clinicians refine hypotheses for SEEG implantation, particularly in diagnostically challenging FCD cases, including MRI-negative patients. However, clinical implementation will require several additional steps. First, the model should be validated prospectively in consecutively enrolled patients. Second, independent multicenter external validation is needed to assess robustness across institutions, EEG acquisition systems, electrode montages, and clinical populations. Third, the framework should be integrated with existing presurgical evaluation data, including MRI, PET, SPECT, seizure semiology, neuropsychological findings, and expert EEG interpretation, rather than being used as a standalone diagnostic tool. Finally, future studies should evaluate whether model-assisted interpretation meaningfully changes clinical decisions, such as SEEG implantation planning or surgical targeting, and whether these changes are associated with improved postoperative seizure outcomes. These steps will be essential for determining the true translational value of ictal scalp EEG-based machine learning in presurgical epilepsy evaluation.

Nonetheless, several limitations must be acknowledged. First, the reliance on expert-annotated seizure onset may introduce inter-rater variability, particularly for ambiguous events. Second, while scalp EEG is noninvasive and widely available, its spatial resolution is inherently limited compared to intracranial recordings. Third, the focus on FCD patients, while clinically relevant, limits direct generalization to other epilepsy etiologies. Fourth, due to the limited sample size, this study categorized seizure localization as temporal versus extratemporal; however, future studies leveraging larger datasets may enable more fine-grained lobe-specific localization algorithms, potentially enhancing clinical utility. Finally, although PCA proved effective in enhancing performance, its interpretability remains limited compared to anatomically grounded features, warranting future research into more explainable representation methods.

In summary, this study presents an interpretable and data-driven pipeline for lateralization and localization of seizure onset zones using ictal scalp EEG. By combining time-frequency analysis, robust ML techniques, and expert clinical input, the proposed framework offers a viable foundation for noninvasive decision support in presurgical epilepsy evaluation.

## Data Availability

The datasets analyzed in this study are not publicly available because of institutional and ethical restrictions related to patient privacy. De-identified data supporting the findings may be available from the corresponding authors upon reasonable request and subject to institutional and ethical approval.
