# Correction: Machine learning-based lateralization and localization of seizure onset in focal cortical dysplasia patients using ictal scalp EEG

**DOI:** 10.3389/fnins.2026.1956659

**Published:** 2026-08-25

**Authors:** Youmin Shin, Sungeun Hwang, Seung-Bo Lee, Hyoshin Son, Jun-Sang Sunwoo, Sang Kun Lee, Kyung-Il Park, Young-Gon Kim

**Affiliations:** 1 Seoul AI School, Seoul School of Sciences and Technologies University, Seoul, Republic of Korea; 2 Interdisciplinary Program in Bio-engineering, Seoul National University, Seoul, Republic of Korea; 3 Department of Transdisciplinary Medicine, Seoul National University Hospital, Seoul, Republic of Korea; 4 Department of Neurology, Ewha Womans University Mokdong Hospital, Seoul, Republic of Korea; 5 Department of Medical Informatics, Keimyung University School of Medicine, Daegu, Republic of Korea; 6 Department of Neurology, The Catholic University of Korea, Eunpyeong St. Mary's Hospital, Seoul, Republic of Korea; 7 Department of Neurology, Kangbuk Samsung Hospital, Sungkyunkwan University School of Medicine, Seoul, Republic of Korea; 8 Department of Neurology, Seoul National University College of Medicine, Seoul, Republic of Korea; 9 Department of Neurology, Seoul National University Hospital, Seoul, Republic of Korea; 10 Department of Neurology, Seoul National University Hospital Healthcare System Gangnam Center, Seoul, Republic of Korea; 11 Department of Medicine, Seoul National University College of Medicine, Seoul, Republic of Korea; 12 Healthcare AI Research Institute, Seoul National University Hospital, Seoul, Republic of Korea

**Keywords:** artificial intelligence, epilepsy, focal cortical dysplasia, ictal EEG, machine learning, seizure onset zone

Affiliation 7 was erroneously provided as “Department of Neurology, Kangbuk Samsung Hospital, Seoul, Republic of Korea”. The correct affiliation 7 is “Department of Neurology, Kangbuk Samsung Hospital, Sungkyunkwan University School of Medicine, Seoul, Republic of Korea.”

The funder Seoul National University Hospital Research Fund, grant no. 0420253040 to Kyung-Il Park, was erroneously omitted. The correct **Funding** statement reads:

“The author(s) declared that financial support was received for this work and/or its publication. This research was supported by research funding from aSSIST University and a grant of the Korea Health Technology R&D Project through the Korea Health Industry Development Institute (KHIDI), funded by the Ministry of Health and Welfare, Republic of Korea (grant number: RS-2023-00265638). This study was also supported by grant no. 0420253040 from the Seoul National University Hospital Research Fund.”

The original version of this article has been updated.

