# Supplementary material for: Machine learning-based lateralization and localization of seizure onset in focal cortical dysplasia patients using ictal scalp EEG
Source: Front Neurosci. 2026 Jul 30;20:1915535. doi: 10.3389/fnins.2026.1915535 (PMC13469425; doi:10.3389/fnins.2026.1915535)
Supplement: Supplementary file 1 [file Data_Sheet_1.DOCX]

Supplementary Materials

**Supplementary Figure 1. Patient selection process.**

Supplementary Figure 2. Overall analysis pipeline.

Schematic overview of the study workflow, including cohort definition and demographics, non-PCA feature-domain and time-window analyses, PCA-based feature analysis, model interpretation, and subgroup robustness assessment.

**Supplementary Figure 3. SHAP importance plot of connectivity features
(Left: lateralization, Right: localization)**

**Supplementary Figure 4. Temporal dynamics of connectivity features
(Top: lateralization, Bottom: localization).**

**Supplementary Figure 5. SHAP importance plot of PCA-transformed morphology features
(Left: lateralization, Right: localization).**

**Supplementary Figure 6. Temporal dynamics of PCA-transformed morphology features
(Top: lateralization, Bottom: localization).**

| GT_1 | GT_2 | Dataset split | MR positive/negative | Pathology type |
| --- | --- | --- | --- | --- |
| R | P | Develop | positive | FCD type IIb |
| R | T | Develop | positive | FCD type IIIa |
| R | T | Develop | negative | FCD type I |
| R | P | Develop | negative | FCD type I |
| L | T | Develop | negative | FCD type I |
| R | T | Develop | positive | FCD type IIIc |
| R | T | Develop | negative | FCD type I |
| L | T | Develop | positive | FCD type IIIa |
| L | T | Develop | positive | FCD type I+IIb |
| L | T | Develop | positive | FCD type IIIa |
| R | F | Develop | negative | FCD type I |
| L | T | Develop | positive | FCD type IIb |
| R | T | Develop | positive | FCD type IIIa |
| R | T | Develop | negative | FCD type Iib |
| R | T | Develop | negative | FCD type I |
| L | T | Develop | positive | FCD type IIIa |
| R | T | Develop | positive | FCD type I |
| R | T | Develop | positive | FCD type IIIb |
| L | F | Develop | positive | FCD type IIa |
| R | T | Develop | negative | FCD type I |
| L | F | Develop | negative | FCD type I |
| R | T | Develop | positive | FCD type IIIa |
| L | P | Develop | positive | FCD type I |
| R | T | Develop | positive | FCD type IIIc |
| R | T | Develop | negative | FCD type I |
| L | T | Develop | negative | FCD type I |
| R | T | Develop | positive | FCD type IIIc |
| R | T | Develop | positive | FCD type IIIa |
| R | T | Develop | negative | FCD type IIa |
| R | O | Develop | negative | FCD type I |
| R | T | Develop | negative | FCD type I |
| R | T | Develop | positive | FCD type IIIc |
| R | T | Develop | negative | FCD type I |
| R | T | Develop | positive | FCD type IIIc |
| R | F | Develop | positive | FCD type IIa |
| R | F | Develop | negative | FCD type I |
| R | O | Develop | positive | FCD type IIb |
| L | T | Develop | positive | FCD type I |
| R | P | Develop | negative | FCD type I |
| R | T | Develop | negative | FCD type I |
| L | O | Develop | negative | FCD type IIa |
| R | O | Develop | negative | FCD type I |
| R | T | Develop | negative | FCD type I |
| R | T | Develop | positive | FCD type IIIb |
| L | F | Develop | positive | FCD type IIb |
| L | F | Develop | negative | FCD type I |
| R | T | Develop | negative | FCD type IIIb |
| R | T | Develop | positive | FCD type IIb |
| R | T | Develop | positive | FCD type IIIa |
| R | T | Develop | negative | FCD type I |
| L | F | Develop | positive | FCD type IIId |
| R | P | EV | positive | Image FCD |
| R | T | EV | positive | Image FCD |
| L | P | EV | positive | Image FCD |
| R | T | EV | positive | Image FCD |
| R | T | EV | positive | Image FCD |
| L | O | EV | positive | Image FCD |
| L | T | IV | positive | FCD type I |
| R | F | IV | positive | FCD type I |
| R | F | IV | positive | FCD type I |
| R | T | IV | positive | FCD type IIIc |
| R | T | IV | positive | FCD type I |
| R | O | IV | positive | FCD type IIa |
| L | T | IV | positive | FCD type IIIa |
| L | T | IV | positive | FCD type I |
| R | O | IV | negative | FCD type IIId |
| L | P | IV | negative | FCD type I |
| R | T | IV | negative | FCD type I |
| R | T | IV | negative | FCD type IIIa |

Supplementary Table 1. Dataset split, MRI status, and pathology type for FCD cases.

GT_1 indicates lateralization; GT_2 indicates localization. L, left; R, right; T, temporal; F, frontal; P, parietal; O, occipital; IV, internal validation; EV, independent non-surgical validation.

| **Cohort / fold** | **N** | **Left** | **Right** | **Temporal** | **Extratemporal** | **MRI-positive** | **MRI-negative** |
| --- | --- | --- | --- | --- | --- | --- | --- |
| Lateralization cross-validation folds stratified by SOZ laterality | | | | | | | |
| Development fold 1 | 17 | 5 | 12 | 12 | 5 | 11 | 6 |
| Development fold 2 | 17 | 5 | 12 | 13 | 4 | 7 | 10 |
| Development fold 3 | 17 | 5 | 12 | 10 | 7 | 9 | 8 |
| Localization cross-validation folds stratified by SOZ location | | | | | | | |
| Development fold 1 | 17 | 6 | 11 | 11 | 6 | 6 | 11 |
| Development fold 2 | 17 | 4 | 13 | 12 | 5 | 10 | 7 |
| Development fold 3 | 17 | 5 | 12 | 12 | 5 | 11 | 6 |
| Validation cohorts | | | | | | | |
| Internal validation | 12 | 4 | 8 | 7 | 5 | 8 | 4 |
| Extra validation | 6 | 2 | 4 | 3 | 3 | 6 | 0 |

Supplementary Table 2. Distribution of laterality, localization, and MRI status across cross-validation folds and validation cohorts

Abbreviations: MRI, magnetic resonance imaging; SOZ, seizure onset zone.

| Task | Set | AUC | ACC | SEN | SPE |
| --- | --- | --- | --- | --- | --- |
| L vs R | 3-fold | 0.874±0.024 | 0.824±0.014 | 0.867±0.136 | 0.806±0.019 |
|  | Internal | 0.781±0.072 | 0.667±0.115 | 0.750±0.192 | 0.625±0.082 |
|  | Extra | 0.750±0.250 | 0.833±0.166 | 0.500±0.500 | 1.000±0.000 |
| T vs ET | 3-fold | 0.915±0.018 | 0.824±0.025 | 0.902±0.018 | 0.687±0.109 |
|  | Internal | 0.786±0.165 | 0.667±0.192 | 0.571±0.250 | 0.800±0.188 |
|  | Extra | 0.778±0.111 | 0.722±0.096 | 0.444±0.192 | 1.000±0.000 |

Supplementary Table 3. Classification performance metrics at the best-performing window (w/o PCA).

| GT | Feature domain | PCA strategies | AUC |
| --- | --- | --- | --- |
| L vs R | Connectivity | Laterality-wise | 0.635±0.069 |
| L vs R | Connectivity | Lobe-wise | 0.757±0.098 |
| L vs R | Connectivity | Entire-channel-level | 0.629±0.087 |
| L vs R | Morphology | Laterality-wise | 0.648±0.080 |
| L vs R | Morphology | Lobe-wise | 0.616±0.058 |
| L vs R | Morphology | Entire-channel-level | 0.767±0.058 |
| T vs ET | Connectivity | Laterality-wise | 0.650±0.053 |
| T vs ET | Connectivity | Lobe-wise | 0.662±0.093 |
| T vs ET | Connectivity | Entire-channel-level | 0.735±0.080 |
| T vs ET | Morphology | Laterality-wise | 0.661±0.085 |
| T vs ET | Morphology | Lobe-wise | 0.688±0.051 |
| T vs ET | Morphology | Entire-channel-level | 0.796±0.098 |

Supplementary Table 4. Performance comparison of feature domains and PCA strategies

| **Task** | **Set** | **AUC** | **ACC** | **SEN** | **SPE** |
| --- | --- | --- | --- | --- | --- |
| L vs R | 3-fold | 0.880±0.053 | 0.834±0.032 | 0.853±0.011 | 0.765±0.085 |
|  | Internal | 0.813±0.031 | 0.861±0.048 | 0.750±0.000 | 0.917±0.072 |
|  | Extra | 0.750±0.125 | 0.722±0.096 | 0.500±0.000 | 0.833±0.144 |
| T vs ET | 3-fold | 0.890±0.011 | 0.843±0.020 | 0.867±0.031 | 0.820±0.021 |
|  | Internal | 0.800±0.029 | 0.833±0.000 | 0.810±0.083 | 0.867±0.116 |
|  | Extra | 0.833±0.167 | 0.813±0.167 | 0.880±0.192 | 0.723±0.192 |

Supplementary Table 5. Classification performance metrics at the best-performing window (w/ PCA)

| **Task** | **Set** | **AUC** | **ACC** | **SEN** | **SPE** |
| --- | --- | --- | --- | --- | --- |
| L vs R  (w/o PCA) | Internal | 0.781±0.072 | 0.667±0.115 | 0.750±0.192 | 0.625±0.082 |
|  | MR positive | 0.778±0.077 | 0.625±0.111 | 0.778±0.192 | 0.833±0.115 |
|  | MR negative | 0.778±0.192 | 0.583±0.144 | 0.667±0.577 | 0.556±0.192 |
| T vs ET  (w/o PCA) | Internal | 0.786±0.165 | 0.667±0.192 | 0.571±0.250 | 0.800±0.188 |
|  | MR positive | 0.789±0.083 | 0.625±0.125 | 0.533±0.115 | 0.778±0.192 |
|  | MR negative | 0.667±0.144 | 0.583±0.144 | 0.500±0.000 | 0.667±0.289 |
| L vs R  (w/ PCA) | Internal | 0.813±0.031 | 0.861±0.048 | 0.750±0.000 | 0.917±0.072 |
|  | MR positive | 0.799±0.067 | 0.791±0.072 | 1.000±0.000 | 0.667±0.115 |
|  | MR negative | 0.889±0.096 | 0.833±0.144 | 1.000±0.000 | 0.778±0.192 |
| T vs ET  (w/ PCA) | Internal | 0.800±0.029 | 0.833±0.000 | 0.810±0.083 | 0.867±0.116 |
|  | MR positive | 0.800±0.176 | 0.833±0.144 | 0.867±0.115 | 0.778±0.192 |
|  | MR negative | 0.792±0.361 | 0.833±0.289 | 0.833±0.289 | 0.833±0.289 |

Supplementary Table 6. Classification performance metrics for MR-positive and MR-negative subgroups

Performance was evaluated using Youden-index thresholds within the internal validation set.

| **Task** | **Set** | **AUC** | **ACC** | **SEN** | **SPE** |
| --- | --- | --- | --- | --- | --- |
| L vs R  (w/o PCA) | Temporal | 0.833±0.144 | 0.810±0.083 | 0.778±0.193 | 0.833±0.144 |
|  | Extra-temporal | 0.666±0.144 | 0.467±0.116 | 0.667±0.577 | 0.417±0.144 |
| L vs R  (w/ PCA) | Temporal | 0.889±0.192 | 0.905±0.165 | 0.889±0.192 | 0.917±0.144 |
|  | Extra-temporal | 0.583±0.382 | 0.667±0.306 | 1.000±0.000 | 0.583±0.382 |

Supplementary Table 7. Lateralization performance in temporal and extratemporal SOZ subgroups

Performance was evaluated using Youden-index thresholds within the internal validation set.
